# Fake Therapists: How Short Courses Lead to Long-Term Mental Health Issues

**DOI:** 10.1192/j.eurpsy.2025.2150

**Published:** 2025-08-26

**Authors:** E. Molchanova

**Affiliations:** Psychology, American University of Central Asia, Bishkek, Kyrgyzstan

## Abstract

**Introduction:**

In Kyrgyzstan and other Central Asian countries, there has been a surge in advertisements for psychological services from underqualified practitioners. These individuals, often with only short-term training, market themselves as professionals while the cost of their services has risen dramatically. Vulnerable populations may be exploited financially without receiving effective care, leading to worsened outcomes.

**Objectives:**

This paper explores how the rise in underqualified practitioners and unchecked price increases for psychological services in Kyrgyzstan lead to harmful mental health outcomes. It also compares the regulatory frameworks in neighboring countries, highlighting Kazakhstan’s more structured approach.

**Methods:**

This study utilizes a comparative analysis of the regulatory frameworks for psychological services in Kyrgyzstan, Tajikistan, Uzbekistan, and Kazakhstan. Data is sourced from governmental reports, academic studies, and analyses of online advertisements for psychological services. The focus is on licensing systems, training requirements, and the impact of rising consultation prices.

**Results:**

Kyrgyzstan, Tajikistan, and Uzbekistan lack adequate regulatory systems for licensing psychologists. The surge in online advertisements by underqualified practitioners has flooded the market, with many offering their services at increasingly inflated rates. These individuals often charge exorbitant prices while lacking the proper training, exacerbating mental health challenges for vulnerable populations. In contrast, Kazakhstan has introduced a structured National Qualifications Framework (SQF), which ensures that psychological services are provided by properly trained and certified professionals.

**Conclusions:**

The unchecked rise of advertisements by unqualified therapists and the unlimited escalation of consultation fees in Kyrgyzstan and neighboring countries pose serious risks to public mental health. Without proper licensing and price regulation, individuals seeking help may face harmful consequences. Adopting a regulatory system similar to Kazakhstan’s, with strict licensing and certification requirements, would help ensure that psychological services are both safe and affordable. Such reforms are essential to protect the public from the exploitation and risks posed by untrained “therapists.”

**Disclosure of Interest:**

None Declared

